# Benefits of a family-based care transition program for older adults after hip fracture surgery

**DOI:** 10.1007/s40520-024-02794-8

**Published:** 2024-07-13

**Authors:** Sahar Mashhadi-Naser, Fatemeh Pashaei Sabet, Malihe Nasiri, Parvaneh Vasli

**Affiliations:** 1 Student Research Committee, Department of Community Health Nursing, School of Nursing and Midwifery, Shahid Beheshti University of Medical Sciences, Tehran, Iran; 2 Department of Community Health Nursing, School of Nursing and Midwifery, Shahid Beheshti University of Medical Sciences, Tehran, Iran; 3 Department of Basic Sciences, School of Nursing and Midwifery, Shahid Beheshti University of Medical Sciences, Tehran, Iran

**Keywords:** Hip fracture, Older adults, Activities of daily living, Health-related quality of life, Social support

## Abstract

**Background:**

Hip fracture (HF) in older adults is strongly associated with a greater decline in their activities of daily living (ADLs) and health-related (HRQoL). The present study aimed to evaluate the effects of a family-based care transition program (FBCTP) on ADLs, HRQoL and social support in this age group after HF surgery.

**Methods:**

A quasi-experimental design was conducted on 100 older adults who had undergone HFS and were selected by convenience sampling and allocated to the IG (*n* = 50) and the CG (*n* = 50). Data were collected utilizing the Barthel Index, the 12-item Short Form Health Survey (SF-12), and the Multidimensional Scale of Perceived Social Support. The FBCTP was delivered in-hospital education sessions, home visit, and a follow-up and telephone counselling session. The data were collected at three stages, including the baseline, four weeks after discharge, and eight weeks later. The level of statistical significance was set at 0.05.

**Results:**

The results of the study indicated that the effects of time and group on the increase in ADLs were 15.2 and 36.69 (*p* < 0.000), respectively, following the completion of the FBCTP. Furthermore, time and group were found to have a positive effect on HRQoL, with an increase of 2.82 and 5.60 units, respectively (*p* < 0.000). In this context, time and group also interacted in the IG compared to the CG, with scores increasing by 1.86 units over time (*p* < 0.000). Although the study results indicated that social support improved by 1.98 units over time (*p* < 0.000), the effects of group alone and the time × group interaction were not statistically significant. This indicates that the program was not effective in accelerating social support.

**Conclusion:**

Consequently, nurses, policymakers, and planners engaged in geriatric healthcare may utilize these results to enhance the health status of this age group following HFS.

## Introduction

Given the upward trend in the world’s population ageing, there is a high prevalence rate of osteoporosis and its serious consequences, including hip fracture (HF) in older adults [1]. Accordingly, HF has been so far acknowledged as one of the leading causes of disability and mortality in this age group, and its related costs have been a global economic burden. It is notable that fewer than 40–60% of HF survivors regain their pre-fracture mobility status [2]. Furthermore, this condition is closely associated with multiple deficiencies in activities of daily living (ADLs), which subsequently result in loss of independence and low self-esteem. The regression in such activities subsequently results in a number of adverse outcomes, including a decline in quality of life (QoL) and an increase in admissions to nursing homes [3]. A reflection on the health-related quality of life (HRQoL) as a multidimensional concept, denoting a person’s experience of general health with regard to specific dimensions, namely, the physical, social, emotional, and functional ones [4], indicates that the lowest HRQoL seems to ensue during the first three months after hip fracture surgery (HFS) [5].

It is likely that the HRQoL is determined by social support and family, as most patients experiencing HFS go home after discharge and receive care from their family caregivers [6]. Social support is an important factor associated with ADLs in older adults [7]. Social support represents the provision of emotional, instrumental, or informational resources to these individuals in order to manage stress and life events [8]. Furthermore, the social support can have a multitude of positive effects on an individual’s health status, encouraging a series of health-related behaviors, such as taking medication on time, engaging in regular exercise, and controlling one’s diet [7]. To date, some studies have established that lower social support, being alone, and insufficient self-management skills throughout post-discharge care may result in recurrent hospitalizations [9].

Given the importance of supporting such patients to regain mobility and independence after surgery and their hip joint immobilization for a few days [10], the prompt start of ADLs [11], hospital discharge as soon as possible and rehabilitation at home after HFS are in most cases advocated by healthcare facilities [10]. In light of this, continuity of care at home enriches QoL, accelerates functional recovery, and mitigates many side effects [12]. In this context, transition from hospital to home is a multifaceted event associated with many changes in care roles and responsibilities from healthcare providers to family caregivers [13]. Accordingly, care transition has been described as a series of actions planned to ensure coordination and continuity of care for patients from admission to discharge or transfer between wards or healthcare facilities [14]. Inadequate care transition is associated with some adverse outcomes, such as readmissions, prolonged hospital stays and medication errors [15].

Care transition generally occurs in the continuum of discharge planning and management once long-term care is required. While discharge planning is assumed as a process at the point of care (i.e., an operating room or a ward) by a patient reference group and then reviewed by consultants, care transition is a multifaceted function that requires a planned discharge with post-discharge support and follow-up [16]. It also refers to a selection of time-limited interventions with a strong focus on hospital-to-home care to optimize patient-centered performance management [17].

Care transition planning and management for such patients and their family caregivers can shorten hospital stay, reduce readmissions, and even meet their satisfaction and that of healthcare providers, as demonstrated [18]. In this line, family caregivers play a major role in providing home care to patients after undergoing HFS [19]. As a result, family involvement in care transition from hospital to home is an important issue [20]. It is worth noting that family caregivers are actually the family members who establish emotional relationships with patients and can continue to provide emotional support and care during the course of their illness [21]. Accordingly, these family caregivers demand much more information about care and related services to improve walking ability in older adults after HFS [19]. Despite this, family caregivers rarely receive formal training in the same way as healthcare providers, and require certain informational and emotional support from healthcare systems to take on the responsibilities of informal caregivers [22].

## Literature Review

A number of studies have investigated the effects of care transition, care continuity, and at-home visits on a range of variables, including QoL, ADLs, and social support. For instance, Ko et al. [23] examined the impact of a care transition-based IG on physical functioning and QoL in older adults following HFS in South Korea. Additionally, Liu et al. [12] investigated the efficacy of continuous care on postoperative QoL and long-term functional improvement in this age group with the same conditions. Furthermore, the feasibility and safety of an individualized intervention program based on the ADLs for the rehabilitation of the elderly with HF had been explored in another study [24]. In a separate study, the impact of pre-discharge home visits on reducing falls and avoiding readmissions was considered within the first 30 days and six months after discharge in patients living with HF [25]. A review of previous research revealed that no study had examined the effects of a family-based care transition program (FBCTP) on significant health outcomes. For this reason, the present study was designed to investigate the effectiveness of an FBCTP in ADLs, HRQoL, and social support in older adults following HFS. The following hypotheses were thus addressed:

1. The FBCTP has an effect on ADLs in older adults following HFS.
2. The FBCTP has an effect on HRQoL in older adults following HFS.
3. The FBCTP has an effect on social support in older adults following HFS.

## Methods

### Study design and setting

This study employed a quasi-experimental, before-after research design, and was conducted within orthopedic wards serving men and women at Shohaday-Haftom-Tir Hospital, a referral center in Tehran, Iran. The two wards in question had 42 and 40 beds, respectively, with five to seven beds allocated to patients with HF in each ward.

### Participants and recruitment

The study was conducted on a population of older adults who had undergone HFS and then been hospitalized in orthopedic wards. The eligible participants, selected by convenience sampling, were allocated to the intervention group (IG) and the control group (CG) in accordance with the study design. With regard to gender, the participants were selected equally from both male and female wards and included in the study groups.

The inclusion criteria for recruiting older adults were as follows: (1) age 60 or above; (2) hospitalization as a consequence of HF; (3) an family caregivers from admission to home care; (4) residence in the city of Tehran; (5) understanding of the Persian language; (6) literacy in reading and writing for patients or their family caregivers; (7) imminent discharge upon physician’s order in the next three days; (8) possession of a smartphone. The patients and their family caregivers were required to have no severe hearing or vision disorders, not suffer from serious movement disorders prior to HF, not experience chronic systemic diseases, including multiple sclerosis, systemic lupus erythematosus, rheumatoid arthritis, and so on, not struggle with complex mental, cognitive, and physical problems, show a willingness to cooperate, and not face complex malignancies. Conversely, the exclusion criteria included the withdrawal of the patients or their family caregivers from the study, the expiration of the patients or changes in their family caregivers for the duration of the study, and the patient’s relocation outside the city of Tehran.

In accordance with the type-I error (α) of 0.05, the type-II error (β) of 0.20, and the test power (1-β) of 0.60 in the IC and CG, the sample size was estimated to be 43 individuals, based on the following formula and the study by Liu et al. [12]. However, in consideration of the 20% sample attrition, 50 older adults who had undergone HFS were included in both groups.

**Fig. 1:**
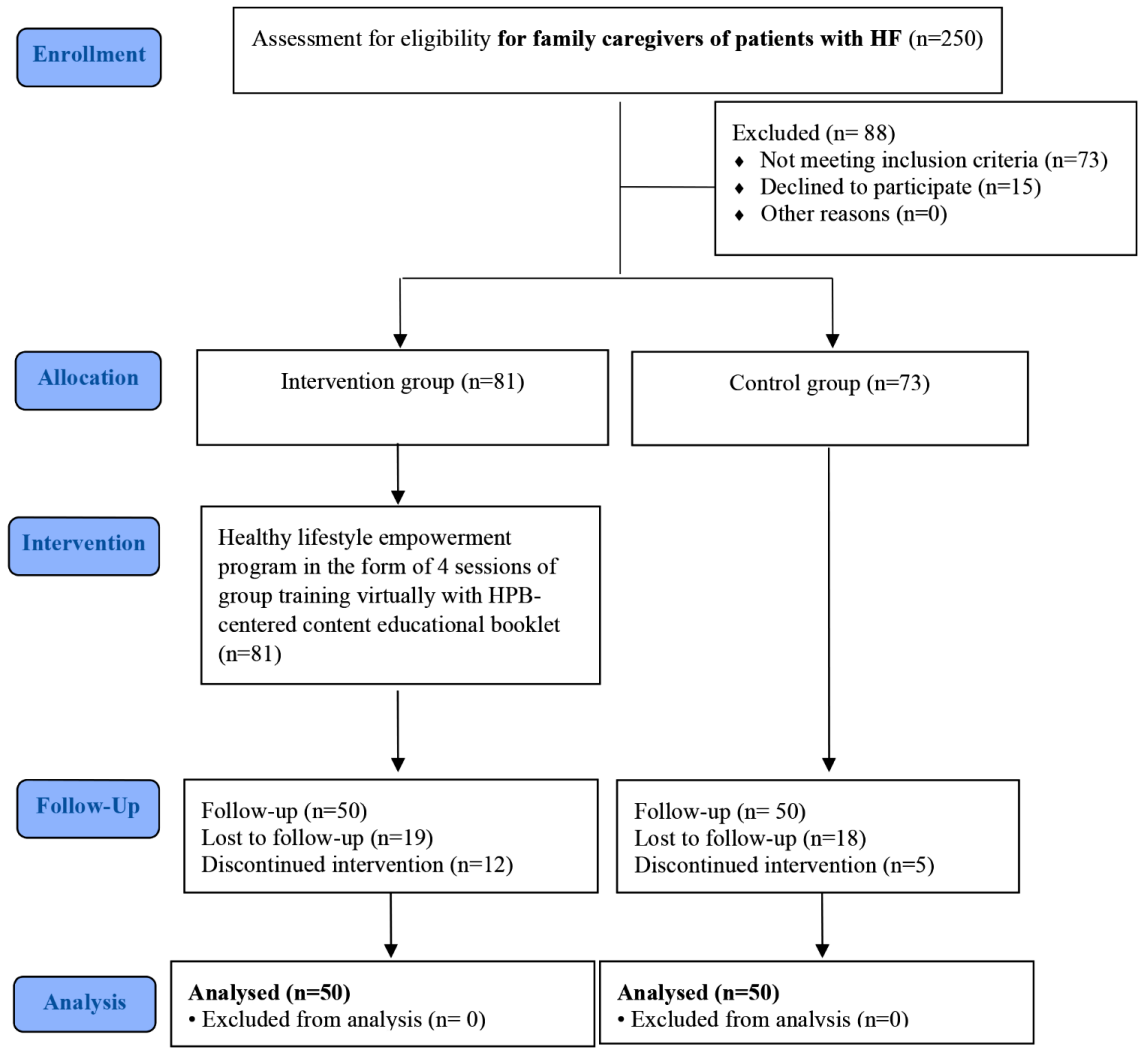
CONSORT flow chart of the study

A total of 154 older adults with the HFS experience were included in the study. At some point during the study, 17 patients were excluded due to their withdrawal from the study, 21 individuals were removed from the study following the development of embolism and heart problems, and 16 cases were not included in the final analysis following readmissions due to surgical site infection, pneumonia, and sepsis. In summary, 100 older adults undergoing HFS (50 in each group) were included in this study (Fig. 1).

### Outcome measurements

This study identified three major outcomes for older adults with HFS: changes in ADLs, HRQoL, and social support, measured at three stages, including the baseline (prior to the FBCTP), follow-up 1 (four weeks after discharge), and follow-up 2 (eight weeks after discharge). The research tools in the follow-up 1 were completed in person and at home. On the same day, the participants were provided with the follow-up 2 research tools, which they were instructed to complete and send to the first researcher via Bale, an Iranian messaging app.

### Measures

Data from the study were collected using four instruments: the Demographic Information Questionnaire, the Barthel Index, the 12-item Short Form Survey (SF-12), and the Multidimensional Scale of Perceived Social Support. Given the advanced age of the HP patients and the potential for limited understanding of the instrument items, all measures were completed by family caregivers after an explanation of the completion process.

### Demographic information questionnaire

The Demographic Information Questionnaire comprised two sections: (a) the demographic information of the patients following HFS, with six items concerning age, gender, marital status, education, job, and number of family members; and (b) the demographic information of the family caregivers, including six items about age, gender, marital status, education, job, and relationship with the patient.

#### Barthel Index

The Barthel Index was employed in this study to assess the capacity of the older patients to perform ADLs. The instrument comprised 10 statements designed to elicit reflections on the participants’ independence or dependence in relation to 10 activities: feeding, bathing, grooming, dressing, bowel control, bladder control, toilet use, transfers from bed to chair and vice versa, and mobility on flat surfaces and stairs. In this context, six activities were scored on a scale of 0, 5, and 10: feeding, dressing, bowel control, bladder control, toilet use, and mobility on stairs. In addition, the values for two activities, namely transfer from bed to chair and vice versa, and mobility on flat surfaces, were 0, 5, 10, and 15, while the other two activities, namely bathing and grooming, were scored 0 and 5. The minimum and maximum values in this questionnaire were thus 0 to 100, respectively. The psychometric properties of the Persian version of the Barthel Index had previously been investigated and confirmed in patients with stroke [26]. The reliability of the instrument was determined using the Cronbach’s alpha method, and after the scale was completed by 20 participants who did not subsequently enter the study, it was 0.78. Considering that the Cronbach’s alpha value above 0.7 is accepted [27], the instrument is accepted in this sense.

#### 12-item short Form Health Survey

The SF-12 was employed to ascertain the HRQoL in both physical and mental domains. It comprised 12 statements distributed across eight dimensions, with the data organized into two subscales: physical and mental. The minimum and maximum scores for this research tool were 12 and 48, respectively. Therefore, the higher the score, the better the HRQoL [13]. The SF-12 has previously been validated and demonstrated to be reliable [28]. In this study, the reliability of this tool was computed using the Cronbach’s alpha method, with a value of 0.75 obtained.

### Multidimensional scale of Perceived Social Support

The Multidimensional Scale of Perceived Social Support was developed by Zimet et al. (1988) for the purpose of measuring perceived social support. The questionnaire comprised 12 items, each based on a five-point Likert-type scale from “Never” (score 1) to “Always” (score 5). The sub-scales included family, friends, and significant others. Furthermore, the minimum and maximum scores were 12 and 60, respectively. The validity and reliability of this tool had been previously established and confirmed [29]. In the present study, the reliability of the scale was determined by the Cronbach’s alpha method, with a value of 0.91.

### Intervention: family-based care transition program

The FBCTP was implemented on the older adults following HFS in the presence of their family caregivers, three days prior to discharge. The program’s content aimed to enhance three variables in this study: ADLs, HRQoL, and social support, through two in-hospital sessions, one education session at home with face-to-face consultation, and one telephone counselling session. The program focused on the following topics: hip anatomy, HF and its treatments, healthy eating, stress management, personal relationships and the use of social media to strengthen social connections, taking medications at home, beginning ADLs and mobility (sitting and standing, bathing, grooming, dressing, bowel and bladder control, toileting, transferring from bed to chair and vice versa, and mobility on flat surfaces and stairs, sleeping correctly), exercises after HFS, use of mobility aids for walking (walker, underarm crutches), advanced mobility based on the patient’s progress (sitting in a car seat, driving, returning to work), assessment of mobility progress. Each session lasted between 20 and 40 min (Table 1).

**Table 1:** FBCTP for older adults after HFS

Sessions	Time	Place	Procedure	Content
First	One-two days before discharge	Inpatient ward	Face-to-face education in the presence of family caregivers	- Hip anatomy, HF, and its treatments - Healthy eating - Stress management - Personal relationships and utilization of social media to bolster social connections - Q&A
Second	On discharge day	Inpatient ward	Face-to-face education in the presence of family caregivers	- Review of previous session contents - Ways to take medicines at home - Beginning activities of daily living and mobility (sitting and standing, bathing, grooming, dressing, bowel and bladder control, toileting), (sitting and standing, bathing, personal hygiene, dressing, toileting, transferring from bed to chair and vice versa, and mobility on flat surfaces and stairs, sleeping correctly) - Exercises after HFS, - Q&A
Third	Second week after discharge	Home	Home-visit and face-to-face education in the presence of family caregivers	- Review of previous session contents - Use of mobility aids for walking (walking frame, underarm crutches) - Advanced mobility based on the patient’s progress (sitting in a car seat, driving, returning to work) - Q&A
Fourth	Fourth week after dischargee	Home	Simultaneous telephone counseling with patients and family caregivers	- Review of previous session contents - Assessment of mobility progress, possible side effects, and medicine use - Q&A

*FBCTP*, family-based care transition program; *HFS*, hip fracture surgery; *Q&A*, question and answer

Prior to the implementation of the program, a social messaging application, such as Bale, WhatsApp, or Telegram, was installed on the smartphones of the patients and their family caregivers, if necessary, in order to facilitate the sharing of images of completed research tools with the researcher. It is notable that the participants in the CG only received routine education during discharge. This comprised general information about taking medicines and starting physical activities for 5–10 min.

### Data analysis

Once the data collection phase was complete, the questionnaires were coded and the data were analyzed using the SPSS Statistics software (version 22). Descriptive and inferential statistics were employed with regard to the 95% confidence interval to describe the data, a frequency table and graph were constructed, along with mean, standard deviation, frequency, and percentage. The independent-samples t-test, Chi-square test, and Mann-Whitney U test were employed to facilitate a comparison between the demographic information of the IC and CG. Furthermore, the independent-samples t-test and analysis of covariance (ANCOVA) were employed for the within-group comparison, while the repeated measures ANOVA was utilized for the between-group comparison. Furthermore, a two-way ANOVA was employed to identify the time × group interaction effects on the primary variables in this study.

## Results

### Baseline information of participants

The study results indicated that the mean ± SD age of the older adults following HFS in the IC and CG was 72.40 ± 7.38 and 67.74 ± 6.12, respectively, demonstrating a statistically significant difference (*p* = 0.001). Furthermore, the mean ± SD of the age of the (Table 1) family caregivers in the IC and CG were 49.52 ± 12.43 and 49.84 ± 12.83, respectively, with no significant difference observed. With the exception of the family size of older adults with HFS (*p* = 0.001), no significant differences were observed in the demographic variables related to these individuals and their family caregivers between the IC and CG (Table 2).

**Table 2:** Baseline demographic information of the older adults with HFS in the intervention and control groups

Group Variables		IG (*n*=50)		IC (*n*=50)		p-value
		Frequency	Percentage	Frequency	Percentage	
Patients	Gender					
	Female	25	50	25	50	-
	Male	25	50	25	50	
	***Marital status***					
	Single	3	6	2	4	0.784^a^
	Married	24	48	29	58	
	Widow	19	38	16	32	
	Divorced	4	8	3	6	
	***Education***					
	Primary and middle school	40	80	45	90	0.225^a^
	High school and diploma	8	16	5	10	
	Academic	2	4	0	0	
	***Job***					
	Homemaker	20	40	19	38	0.290^a^
	Self-employed	9	18	5	10	
	Employee	1	2	0	0	
	Retired	20	40	26	52	
	Unemployed					
	***Number of family members***					
	One	20	40	16	8	0.001^a^
	Two	24	48	22	44	
	Three	5	10	16	32	
	Four	0	0	2	4	
	Five and more	2	4	1	2	
Family caregivers	***Gender***					
	Female	36	72	30	60	0.205^a^
	Male	14	28	20	40	
	***Marital status***					
	Single	8	16	11	22	0.10^a^
	Married	42	84	34	68	
	Widow	0	0	2	4	
	Divorced	0	0	3	6	
	***Education***					
	Primary and middle school	21	42	24	48	0.263^a^
	High school and diploma	17	34	19	38	
	Academic	12	24	7	14	
	***Job***					
	Homemaker	31	62	28	56	0.052^a^
	Self-employed	14	28	7	14	
	Employee	2	4	4	8	
	Retired	3	6	11	22	
	***Relationship with the patient***					
	Spouse	14	28	23	46	0.24^a^
	Child	27	54	18	36	
	Daughter-in-law/son-in-law	4	8	5	10	
	Brother/sister	5	10	4	8	

Note: ^a^ Chi-square test; ^b^ Mann-Whitney U test

*p*< 0.05

*HPS*, hip fracture surgery; *IG*, intervention group; *CG*, control group

### ADLs changes in intervention and control groups

The results of the independent samples t-test indicated that there was no significant difference between the mean scores of the ADLs in the older adults after HFS in the IC and CG at the baseline. However, the ANCOVA outcomes for the follow-ups 1 and 2 demonstrated a significant difference between both study groups (*p* < 0.000). In the within-group comparison, the results of the repeated measures ANOVA demonstrated that the mean scores of the ADLs in the older adults following HFS in the IC and CG exhibited a significant upward trend from the baseline to the follow-up 2 (*p* < 0.000), with a greater increase observed in the IG (Table 3). The two-way ANOVA outcomes demonstrated that time and group, respectively, had an impact on expanding the ADLs by 15.2 and 36.69 (*p* < 0.000), yet they exhibited no interaction effect in this context.

**Table 3:** Comparison of ADLs change in older adults with HFS in the intervention and control groups

Group	IG (*n*=50)	CG (*n*=50)	IG vs. CG ^a^	Interactive effect of time and group			
					B	SD	*p*-value^c^
Baseline	24.80±15.45	19.10±12.40	0.045	Time	15.2	0.94	< 0.001
Follow-up 1	72.10±9.26	37.90±10.74	< 0.001	Group	36.69	4.34	< 0.001
Follow-up 2	85.10±4.22	53.10±10.88	< 0.001	Time*Group	2.20	1.54	0.152
Follow-ups vs. Baseline^b^	< 0.001	< 0.001					

Note: data are represented as mean±standard deviations; ^a^ p-values for comparing scores between the intervention and control groups, at baseline (derived from independent t-test) and at follow-ups (derived from ANCOVA), ^b^ p-value for comparing differences between follow-ups and baseline (derived from repeated measures ANOVA), ^c^ p-value for testing the effect of time and group on the research variables (derived from two-way ANOVA)

*p*< 0.05

*ADLs*, activities daily living; *HFS*, hip fracture surgery; *IG*, intervention group; *CG*, control group

### HRQoL changes in intervention and control groups

As indicated in Table 4, the independent-samples t-test revealed no significant difference in the mean scores of the HRQoL in the older adults following HFS in the IC and CG at the baseline. However, the ANCOVA outcomes at the subsequent two stages, follow-ups 1 and 2, demonstrated a significant difference between both study groups (*p* < 0.000). The results of the within-group comparison indicated that the mean scores of the HRQoL and its physical and mental states significantly augmented from the baseline to follow-up 2 (*p* < 0.000), with the IG exhibiting higher scores. The two-way ANOVA results further suggested that the HRQoL exhibited a time-dependent increase of 2.82 units (*p* < 0.000). Furthermore, the group factor had a significant effect on HRQoL, with a rise of 5.60 units in the IG compared to the CG (*p* < 0.000). Similarly, time and group exhibited an interaction effect on HRQoL, indicating that the growing trend in scores over time was greater in the IG than CG by 1.86 units (*p* < 0.000).

**Table 4:** Comparison of HRQoL change in older adults with HFS in the intervention and control groups

Group		IG (*n*=50)		CG (*n*=50)	IG vs. CG ^a^	Interactive effect of time and group			
							B	SD	p-value^c^
Total	Baseline	22.20±4.26		21.4±4.22	0.174	Time	2.82	0.23	<0.001
	Follow-up 1	34.18±3.57		24.14±3.84	<0.001	Group	5.06	1.18	<0.001
	Follow-up 2	38.86±2.79		26.96±3.54	<0.001	Time*Group	1.86	0.47	<0.001
	Follow-ups vs. Baseline^b^	< 0.001		< 0.001					
Psychical	Baseline	8.74±1.93		8.54±1.51	0.133				
	Follow-up 1	14.20±1.78		10.48±1.40	<0.001				
	Follow-up 2	10.48±1.40		16.70±1.50	<0.001				
	Follow-ups vs. Baseline^b^	< 0.001		< 0.001					
Mental	Baseline	13.46±2.97		12.50±3.36	0.566				
	Follow-up 1	19.98±2.55		13.66±3.08	<0.001				
	Follow-up 2	22.16±1.84		14.88±2.92	<0.001				
	Follow-ups vs. Baseline^b^	< 0.001	< 0.001						

Note: data are represented as mean±standard deviations; ^a^ p-values for comparing scores between the intervention and control groups, at baseline (derived from independent t-test) and at follow-ups (derived from ANCOVA), ^b^ p-value for comparing differences between follow-ups and baseline (derived from repeated measures ANOVA), ^c^ p-value for testing the effect of time and group on the research variables (derived from two-way ANOVA)

*p*< 0.05

*HRQoL*, health-related quality of life; *HFS*, hip fracture surgery; *IG*, intervention group; *CG*, control group

### Social support changes in intervention and control groups

A comparison of the mean scores of social support at all three stages between the IC and CG, as presented in Table 5, revealed that the independent-samples t-test and ANOVA results indicated a higher mean score for social support in the IG than in the CG (*p* < 0.05). With regard to the family dimension, the social support mean scores at the baseline and follow-up 1 were not significantly different in both study groups. However, this was higher in the IG than CG during the follow-up 2 (*p* = 0.01). While the mean scores of social support in the friends dimension at the baseline and follow-up 1 were higher in the IG than CG (*p* < 0.05), there was no significant difference in the follow-up 2. In the dimension of significant others, the mean scores at the baseline were not significantly different in both study groups, but such values in the follow-ups 1 and 2 were higher in the IG than CG (*p* < 0.05). The results of the within-group comparison demonstrated that the mean scores of social support and all its three dimensions exhibited a significant improvement from the baseline to follow-up 2 in both groups (*p* < 0.000). However, the improvement was more pronounced in the IG. The two-way ANOVA outcomes indicated that social support was enhanced by 1.98 over time (*p* < 0.000), but the group separately and the time × group interaction did not have a significant effect on this variable. This implies that the desired FBCTP was ineffective in increasing social support.

**Table 5:** Comparison of social support change in older adults with HFS in the intervention and control groups

Group		IG (*n*=50)	CG (*n*=50)	IG vs. CG ^a^	Interactive effect of time and group			
						B	SD	p-value^c^
Total	Baseline	38.10±7.94	34.01±9.27	0.02	Time	1.98	0.18	< 0.001
	Follow-up 1	42.34±6.73	37.54±8.96	0.018	Group	1.07	0.84	0.202
	Follow-up 2	44.36±5.98	39.52±8.76	0.006	Time*Group	0.14	0.35	0.68
	Follow-ups vs. Baseline^b^	< 0.001	< 0.001					
Family	Baseline	14.30±2.76	12.90±4.17	0.051				
	Follow-up 1	15.26±2.41	13.88±3.98	0.299				
	Follow-up 2	15.87±2.11	14.22±3.80	0.01				
	Follow-ups vs. Baseline^b^	< 0.001	< 0.001					
Friends	Baseline	10.24±3.13	7.96±3.28	<0.001				
	Follow-up 1	11.86±2.63	9.38±3.21	0.024				
	Follow-up 2	12.58±2.35	10.34±3.09	0.075				
	Follow-ups vs. Baseline^b^	< 0.001	< 0.001					
Significant others	Baseline	13.56±3.38	13.14±4.26	0.586				
	Follow-up 1	15.22±2.67	14.28±3.84	0.008				
	Follow-up 2	15.88±1.97	14.96±3.64	0.002				
	Follow-ups vs. Baseline^b^	< 0.001	< 0.001					

Note: data are represented as mean±standard deviations; ^a^ p-values for comparing scores between the intervention and control groups, at baseline (derived from independent t-test) and at follow-ups (derived from ANCOVA), ^b^ p-value for comparing differences between follow-ups and baseline (derived from repeated measures ANOVA). ^c^ p-value for testing the effect of time and group on the research variables (derived from two-way ANOVA)

*p*< 0.05

*HFS*, hip fracture surgery; *IG*, intervention group; *CG*, control group

## Discussion

The findings of the study indicated that the FBCTP could enhance ADLs and HRQoL in older adults following HFS, yet had no impact on the changes in social support. Consequently, only two research hypotheses were confirmed.

With regard to the first hypothesis, the study results indicated that the FBCTP could enhance the ADLs of older adults following HFS. Other studies had also reported similar findings. In a similar vein, Liu et al. [12] had demonstrated that post-discharge care continuity could effectively increase post-operative hip joint function in such patients. In a further investigation, the results indicated that exercise-based interventions could enhance physical performance and mobility in elderly individuals with HF [30]. In a separate study, home visits were found to enhance functional independence in patients with HF six months after discharge [25]. Despite comparable results in other studies, Ko et al. [23] concluded that the care transition program had failed to improve the physical performance of older adults following HFS, including walking and ADLs. The discrepancy between the two studies can be attributed to the differing contents of the care transition programs. The study, which employed an intervention with distinct content from other studies, yielded comparable outcomes. Consequently, it can be posited that the implementation of a FBCTP, wherein the active participation of family caregivers in the care transition is encouraged, can enhance the ADLs of older adults with HFS.

In relation to the second hypothesis, the study results demonstrated that the FBCTP was efficacious in enhancing the HRQoL of older adults following HFS. In line with these findings, Ko et al. [23] additionally demonstrated that the care transition program had enhanced QoL in older adults following HFS. In a separate study, the results indicated that continuity of care after discharge could enhance postoperative QoL in elderly individuals with HF [12]. Although the results of the present study indicated that the FBCTP was effective in improving the HRQoL of older adults after HFS, different outcomes were observed in another study in which an 18-month community-based exercise, osteoporosis education, and behavior change program failed to improve the HRQoL of older adults at risk of fracture [31]. One of the potential explanations for the observed differences in outcomes is the continuation of the program through telephone consultations and home visits, which have been shown to improve HRQoL within 8 weeks.

The results of the study did not confirm the third hypothesis, indicating that the FBCTP was unable to enhance social support in older adults following HFS. These findings are at odds with those of Li et al. [32], who demonstrated that comprehensive social support interventions, including health education, psychotherapy, and family and community support, could enhance social support in elderly individuals living with tuberculosis, compared to basic health education. In a separate study, the Texercise Select program was employed as an intervention, resulting in a further increase in self-efficacy and perceived social support in older adults with regard to their physical activity [33]. Additionally, Czaja et al. [8] demonstrated that the utilization of a computer system designed for this age group could reduce perceived vulnerability, social isolation, loneliness, and QoL. A meta-review indicates that the most successful interventions include social cognitive training programs delivered via telehealth, which enable social interactions based on education and access to information and communication technology. Other successful interventions include social support improvement via online support groups, access to social robots and virtual pets, and digital infrastructure, together with other smart technologies. These interventions could be a support for older adults [34]. The primary reason for the ineffectiveness of the FBCTP in promoting social support in this study was its content, time, and implementation procedure. Therefore, it was essential to adapt and complement this program.

This study is significant in two respects. First, it focuses on a vulnerable group of society, namely older adults following HFS, measures the effects of a feasible multifaceted intervention on two important health-related variables, namely ADLs and HRQoL, and second, that family caregivers were actively involved in receiving and implementing the FBCTP components.

## Conclusion

The findings of the study indicated that the desired FBCTP could enhance the ADLs and HRQoL of older adults following the implementation of HFS. In light of these findings, healthcare professionals can utilize this program as a viable and cost-effective approach to enhance physical and mental well-being in older adults following HFS in hospital and at home. Furthermore, nursing planners and managers involved in geriatric healthcare are advised to utilize this program and its comprehensive content as a performance evaluation indicator for wards and healthcare professionals, with a view to accreditation. Given that the FBCTP in this study did not result in any change to social support, it is recommended that the program contents be adapted based on family and community support interventions in order to increase the participation of older adults following HFS. It is also recommended that the effects of the program on social support for this age group be re-examined in further research. Although this study was conducted in Iran, its findings may be applicable to other countries.

## Data Availability

No datasets were generated or analysed during the current study.
